# Prevalence of smoking and postoperative outcomes in people undergoing coronary artery bypass grafting: a UK registry analysis

**DOI:** 10.1111/anae.16525

**Published:** 2025-01-06

**Authors:** Emma Sewart, Alexander Isted, Kitty H. F. Wong, Gudrun Kunst, Ronelle Mouton

**Affiliations:** ^1^ Bristol Medical School University of Bristol UK; ^2^ King's College Hospital NHS Foundation Trust London UK

Tobacco smoking is the leading behavioural risk factor for cardiovascular disease and may double the risk of long‐term mortality after coronary artery bypass grafting (CABG) [1, 2]. Smoking cessation interventions, which combine pharmacological treatment and behavioural support, are effective at supporting abstinence at the time of surgery and at 12 months postoperatively [3]. However, smoking remains more prevalent among those patients undergoing surgery (25%) than in the general UK population (13%) [4, 5]. There is limited contemporary evidence about the burden of smoking in cardiac surgery. This study investigated the prevalence of smoking in people undergoing CABG surgery in the UK and the impact of smoking on postoperative outcomes. This will help with risk assessment of such patients, as well as resource allocation and strategic planning for addressing smoking‐related issues in surgical contexts.

Permission was obtained for the National Institute for Cardiovascular Outcomes Research (NICOR) to release depersonalised patient data from the National Adult Cardiac Surgery Audit (NACSA) under an agreement between NHS England/GIG Cymru and King's College Hospital NHS Foundation Trust. The full process of data submission and processing by NICOR is described elsewhere [6]. All adults undergoing elective CABG between January 2012 and December 2022 were included. Other elective procedures were included only if performed in addition to CABG. Patients were not included if they underwent non‐elective surgery, had no documented smoking status or had chosen not to have their data used for research. This study involved analysis of existing non‐identifiable patient data and was, therefore, exempt from NHS ethics committee approval.

The primary outcome was prevalence of current smoking (one or more cigarettes per day); former smoking (not smoked within the last month); and non‐smoking (never smoked) at the time of surgery. The secondary outcomes were trends in smoking prevalence over time; in‐hospital mortality; postoperative duration of hospital stay; and complications. Smoking status was reported by year of operation and trends in smoking prevalence over time were assessed using multivariable logistic regression, adjusted for age, sex and procedure type. The incidence of postoperative complications and duration of hospital stay were compared between smoking status groups using multivariable logistic regression and Cox proportional hazards regression, respectively, adjusted for surgical risk using the European System for Cardiac Operative Risk Evaluation 2 (EuroSCORE 2) [7]. Statistical analysis was performed using R version 4.2.1 (R Studio, Vienna, Austria) with a two‐sided significance level set at p < 0.05. Multiple imputation was performed using the multivariate imputation by chained equations package (version 4.2.3) to account for missing data with < 50% of missing values. Sensitivity analysis was done using complete case analysis.

A total of 96,071 patients were included in the analysis. Of these, 8237 (8.6%) were current smokers, 52,074 (54.2%) former smokers and 35,760 (37.2%) non‐smokers. Patient characteristics and procedural details by smoking status are detailed in online Supporting Information Table S1. The prevalence of smoking remained stable at 8.6% through the study period (Fig. 1). However, the adjusted odds of smoking were marginally lower with each advancing year (OR 0.99, 95%CI 0.98–1.00, p = 0.03). The proportion of ex‐smokers fell over this period from 56.3% to 49.0% (OR 0.97, 95%CI 0.97–0.98, p < 0.01), while the proportion of non‐smokers increased from 35.0% to 42.4% (OR 1.03, 95%CI 1.03–1.04, p < 0.01). Smokers had higher odds of developing deep sternal wound infections and were more likely to need surgical debridement than non‐smokers (Table 1). No significant differences were observed in the odds of in‐hospital mortality, returning to the operating theatre, developing a new neurological deficit or renal replacement therapy postoperatively between smokers and non‐smokers. The mean duration of hospital stay was slightly shorter for smokers than for non‐smokers (hazard ratio 0.94, 95%CI 0.92–0.97), but no significant differences were observed in outcomes. Complete case analysis result estimates (online Supporting Information Table S2) were almost identical to those of the analysis using imputed data.

**Figure 1:**
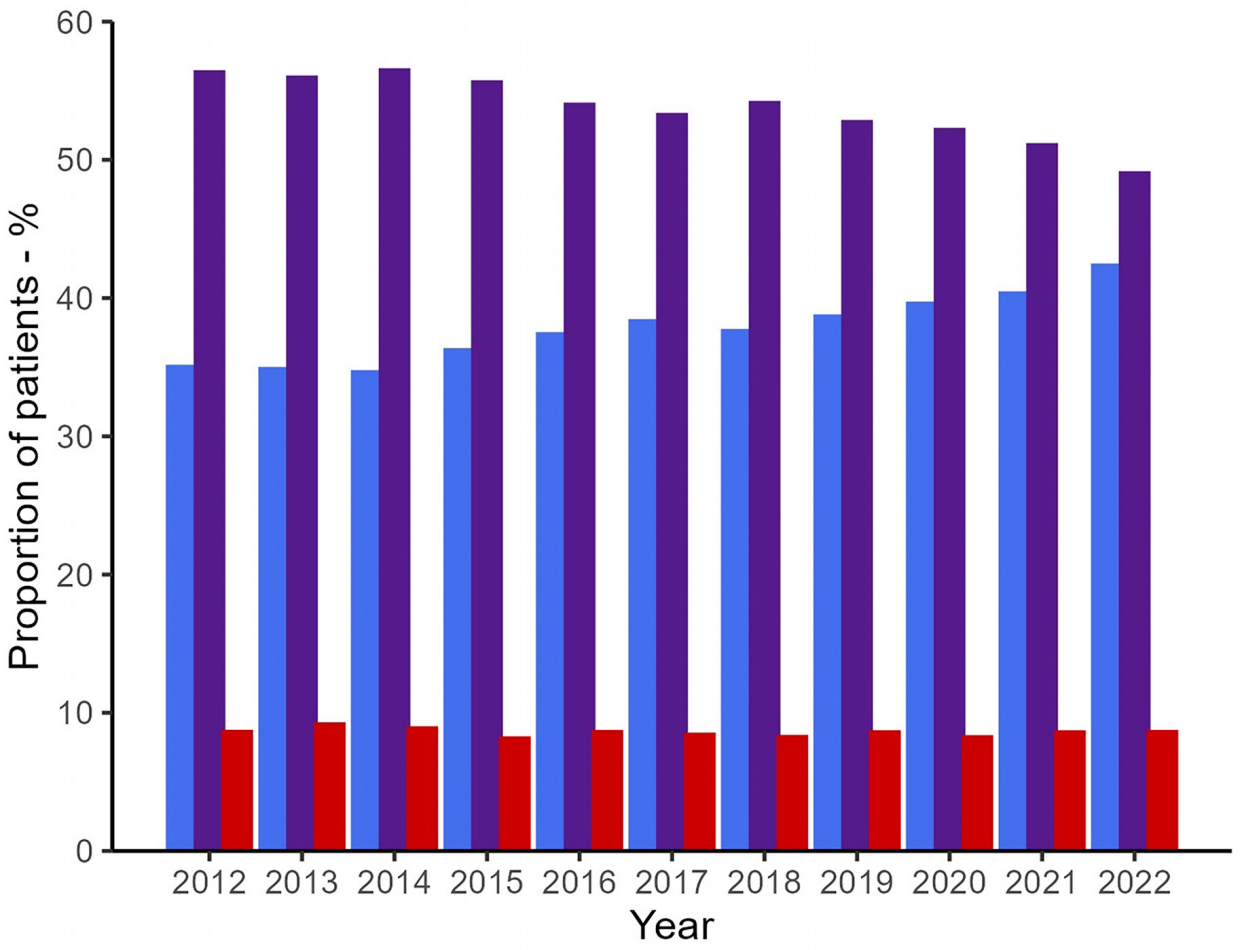
Proportion of non‐smokers (blue), ex‐smokers (purple) and smokers (red) undergoing elective coronary artery bypass grafting per year in the UK from 2012 to 2022.

**Table 1:** Results of adjusted regression models comparing postoperative outcomes using imputed datasets.

	Smokers vs. non‐smokers		Smokers vs. former smokers	
	n = 43,997		n = 60,311	
	OR (95%CI)	p value	OR (95%CI)	p value
In‐hospital mortality	0.97 (0.78–120)	0.76	0.87 (0.71–1.07)	0.19
Return to operating theatre	0.99 (0.87–1.13)	0.93	1.00 (0.88–1.14)	0.96
Deep sternal wound infection				
Any	1.42 (1.08–1.86)	0.01	1.13 (0.87–1.47)	0.35
Requiring surgical debridement	1.86 (1.26–2.77)	< 0.01	1.25 (0.88–1.77)	0.22
New postoperative neurological dysfunction	1.10 (0.89–1.35)	0.39	1.09 (0.88–1.34)	0.43
New postoperative haemofiltration or dialysis	0.92 (0.76–1.11)	0.36	0.86 (0.71–1.03)	0.11

The main limitations of this study were self‐reported smoking status and lack of granularity in the recorded smoking histories, with no stratification for number of cigarettes smoked or timing of cessation. Several important lifestyle risk factors and outcomes of interest, such as long‐term mortality and pulmonary complications, were not available from the NACSA. Finally, some adults undergoing elective CABG in the UK may not be included in the NACSA, but this capture rate is not known.

In this nationwide cohort study, smoking was less prevalent among people undergoing CABG surgery than in the UK general population and other surgical cohorts within the study period. Future work should explore the patterns in smoking behaviour among people referred for cardiac surgery and the factors which support successful quitting in this cohort. Sharing learning with other surgical specialities may improve smoking cessation support within other peri‐operative pathways and allow replication of this rare success story.

## Supporting information


**Table S1.** Patient and procedural characteristics of patients undergoing elective coronary artery bypass graft surgery in the UK between 2012 and 2022, stratified by smoking status.
**Table S2.** Results of adjusted regression models comparing postoperative outcomes in smokers vs. non‐smokers and smokers vs. former smokers using complete cases analysis.
