# Supplementary material for: Prevalence of smoking and postoperative outcomes in people undergoing coronary artery bypass grafting: a UK registry analysis
Source: Anaesthesia. 2025 Jan 6;80(3):330–2. doi: 10.1111/anae.16525 (PMC11825215; doi:10.1111/anae.16525)
Supplement: Supplementary file 1 — Table S1. Patient and procedural characteristics of patients undergoing elective coronary artery bypass graft surgery in the UK between 2012 and 2022, stratified by smoking status. Table S2. Results of adjusted regression models comparing postoperative outcomes in smokers vs. non‐smokers and smokers vs. former smokers using complete cases analysis. [file ANAE-80-330-s001.docx]

**Table S1** Patient and procedural characteristics of patients undergoing elective coronary artery bypass graft surgery in the UK between 2012 and 2022, stratified by smoking status.

|  | **All patients (n = 96,071)** | | **Non-smokers (n = 35,760; 37.2%)** | | **Former smokers (n = 52,074; 54.2%)** | | **Current smokers (n=8,237; 8.6%)** | | ***p* value** | **Missing values** | |
| --- | --- | --- | --- | --- | --- | --- | --- | --- | --- | --- | --- |
| **Age, mean (sd)** | 67.9 +- 9.5 | | 68.4 +- 9.6 | | 68.5 +- 9.1 | | 61.5 +- 9.5 | | <0.01 | 0 | (0.0) |
| **Male sex** | 78,612 | (81.8) | 27,073 | (75.7) | 44,822 | (86.1) | 6,717 | (81.5) | <0.01 | 36 | (0.0) |
| **BMI (kg/m^2^), mean (sd)** | 29.4 +- 10.8 | | 29.1 +- 11.8 | | 29.7 +- 10.1 | | 28.9 +- 10.2 | | <0.01 | 3,741 | (3.9) |
| **Chronic lung disease** | 11,148 | (11.6) | 2,724 | (7.6) | 6,681 | (12.8) | 1,743 | (21.2) | <0.01 | 1,720 | (1.8) |
| **Extracardiac arteriopathy** | 10,499 | (10.9) | 2,213 | (6.2) | 6,822 | (13.1) | 1,464 | (17.8) | <0.01 | 74 | (0.1) |
| **Poor mobility** | 1,590 | (1.7) | 519 | (1.5) | 901 | (1.7) | 170 | (2.1) | <0.01 | 59,644 | (62.1) |
| **Previous cardiac surgery** | 1,747 | (1.8) | 624 | (1.7) | 1,003 | (1.9) | 120 | (1.5) | <0.01 | 4,563 | (4.7) |
| **Active endocarditis** | 46 | (0.0) | 15 | (0.0) | 29 | (0.1) | 2 | (0.0) | 0.39 | 72,010 | (75.0) |
| **Critical preoperative state**** | 823 | (0.9) | 259 | (0.7) | 454 | (0.9) | 110 | (1.3) | <0.01 | 19,601 | (20.4) |
| **Pre-operative creatinine clearance, mean (sd)** | 86.5 +- 32.5 | | 83.7 +- 31.9 | | 86.8 +- 31.9 | | 97.1 +- 36.1 | | <0.01 | 7,543 | (7.9) |
| **Pre-operative renal failure** |  | | | | | | | | 0.06 | 1,235 | (1.3) |
| *Acute renal failure, no dialysis* | 208 | (0.2) | 72 | (0.2) | 116 | (0.2) | 20 | (0.2) |  | | |
| *Dialysis* | 815 | (0.8) | 340 | (1.0) | 396 | (0.8) | 79 | (1.0) |  |  |  |
| **Diabetes** |  | | | | | | | | <0.01 | 208 | (0.2) |
| *Diabetes, not on insulin* | 22,141 | (23.0) | 7,525 | (21.0) | 12,790 | (24.6) | 1,826 | (22.2) |  | | |
| *On insulin* | 6,900 | (7.2) | 2,491 | (7.0) | 3,736 | (7.2) | 673 | (8.2) |  |  |  |
| **CCS angina class IV** | 4,060 | (4.2) | 1,310 | (3.7) | 2,239 | (4.3) | 511 | (6.2) | <0.01 | 98 | (0.1) |
| **Left ventricular function** |  | | | | | | | | <0.01 | 646 | (0.7) |
| *Good (LVEF >50%)* | 73,325 | (76.3) | 28,602 | (80.0) | 38,974 | (74.8) | 5,749 | (69.8) |  | | |
| *Moderate (LVEF 31-50%)* | 19,170 | (20.0) | 6,137 | (17.2) | 10,999 | (21.1) | 2,034 | (24.7) |  |  |  |
| *Poor (LVEF 21-30%)* | 2,803 | (2.9) | 780 | (2.2) | 1,644 | (3.2) | 379 | (4.6) |  |  |  |
| *Very poor (LVEF <=20%)* | 127 | (0.1) | 34 | (0.1) | 77 | (0.1) | 16 | (0.2) |  |  |  |
| **Recent MI (within 90 days)** | 7,373 | (7.7) | 2,460 | (6.9) | 3,951 | (7.6) | 962 | (11.7) | <0.01 | 1,329 | (1.4) |
| **Pulmonary hypertension** |  | | | | | | | | <0.01 | 66,458 | (69.2) |
| *Moderate (PA systolic 31-55mmHg)* | 3,660 | (3.8) | 1,377 | (3.9) | 2,062 | (4.0) | 221 | (2.7) |  | | |
| *Severe (PA systolic >55mmHg)* | 1,256 | (1.3) | 500 | (1.4) | 653 | (1.3) | 103 | (1.3) |  |  |  |
| **NYHA class** |  | | | | | | | | <0.01 | 896 | (0.9) |
| *I* | 19,170 | (20.0) | 8,039 | (22.5) | 9,464 | (18.2) | 1,667 | (20.2) |  | | |
| *II* | 49,177 | (51.2) | 18,213 | (50.9) | 26,838 | (51.5) | 4,126 | (50.1) |  |  |  |
| *III* | 25,244 | (26.3) | 8,621 | (24.1) | 14,411 | (27.7) | 2,212 | (26.9) |  |  |  |
| *IV* | 1,584 | (1.6) | 476 | (1.3) | 947 | (1.8) | 161 | (2.0) |  |  |  |
| **EuroSCORE II (%), mean (sd)** | 2.1 +- 2.5 | | 2.1 +- 2.6 | | 2.1 +- 2.6 | | 1.8 +- 2.3 | | <0.01 | 0 | (0.0) |
| **Weight of Operation** |  |  |  |  |  |  |  |  | <0.01 | 4,829 | (5.0) |
| *Isolated CABG* | 67,181 | (69.9) | 24,476 | (68.4) | 36,361 | (69.8) | 6,344 | (77.0) |  |  |  |
| *Two major procedures* | 20,726 | (21.6) | 7,993 | (22.4) | 11,409 | (21.9) | 1,324 | (16.1) |  |  |  |
| *Three major procedures* | 3,335 | (3.5) | 1,411 | (3.9) | 1,669 | (3.2) | 255 | (3.1) |  |  |  |
| **CABG plus valve surgery** | 29,559 | (30.8) | 10,250 | (28.7) | 14,089 | (27.1) | 1,656 | (20.1) | <0.01 | 1,701 | (1.8) |
| **CABG plus major aortic surgery** | 2,256 | (2.3) | 933 | (2.6) | 1,133 | (2.2) | 190 | (2.3) | <0.01 | 5,266 | (5.5) |

Data are presented as n (%) unless otherwise specified. CCS = Canadian Cardiovascular Society; LVEF = left ventricular ejection fraction; NYHA = New York Heart Association; CABG = coronary artery bypass grafting.

**Table S2** Results of adjusted regression models comparing postoperative outcomes in smokers vs. non-smokers and smokers vs. former smokers using complete cases analysis

|  | **Smokers vs non-smokers (n = 43,997)** | | | | | | **Smokers vs former smokers**  **(n = 60,311)** | | | | | |
| --- | --- | --- | --- | --- | --- | --- | --- | --- | --- | --- | --- | --- |
|  | **OR** | **95% CI** | | **p-value** | **Missing** | | **OR** | **95% CI** | | **p-value** | **Missing** | |
| **In-hospital mortality** | 0.97 | 0.78 | 1.20 | 0.76 | 231 | (0.5) | 0.87 | 0.70 | 1.07 | 0.17 | 289 | (0.5) |
| **Return to theatre** | 1.01 | 0.88 | 1.15 | 0.92 | 3,042 | (6.9) | 1.01 | 0.89 | 1.15 | 0.85 | 5,023 | (8.3) |
| **Deep sternal wound infection** |  |  |  |  |  |  |  |  |  |  |  |  |
| *Any* | 1.48 | 1.12 | 1.96 | 0.01 | 9,826 | (22.3) | 1.15 | 0.88 | 1.49 | 0.32 | 14,856 | (24.6) |
| *Requiring surgical debridement* | 1.92 | 1.29 | 2.85 | <0.01 | 9,865 | (22.4) | 1.33 | 0.92 | 1.91 | 0.14 | 14,919 | (24.7) |
| **New post-operative neurological dysfunction** | 1.10 | 0.89 | 1.37 | 0.38 | 3,506 | (8.0) | 1.09 | 0.88 | 1.34 | 0.43 | 4,417 | (7.3) |
| **New haemofiltration or dialysis post-operatively** | 0.91 | 0.75 | 1.11 | 0.36 | 2,147 | (4.9) | 0.85 | 0.70 | 1.03 | 0.09 | 3,078 | (5.1) |
|  | **HR** | **95% CI** | | **p-value** | **Missing** | | **HR** | **95% CI** | | **p-value** | **Missing** | |
| **Post-operative length of stay (days), mean (SD)** | 0.94 | 0.92 | 0.96 | <0.01 | 382 | (0.9) | 0.98 | 0.96 | 1.00 | 0.07 | 668 | (1.1) |

Logistic regression used for binary outcome variables and Cox proportional hazards used for post-op length of stay. All models using imputed datasets and adjusted for EuroSCORE II. OR = adjusted odds ratio; CI = confidence interval; SD = standard deviation; HR = hazard ratio.
